# Further Evidence of a Specific Psychopathology of Addiction. Differentiation from Other Psychiatric Psychopathological Dimensions (Such as Obesity)

**DOI:** 10.3390/ijerph14080943

**Published:** 2017-08-21

**Authors:** Angelo G.I. Maremmani, Luca Cerniglia, Silvia Cimino, Silvia Bacciardi, Luca Rovai, Alessandro Pallucchini, Vincenza Spera, Giulio Perugi, Icro Maremmani

**Affiliations:** 1 Department of Psychiatry, North-Western Tuscany Region Local Health Unit, Versilia Zone, 55049 Viareggio, Italy; angelogimaremmani@gmail.com; 2 AU-CNS, Association for the Application of Neuroscientific Knowledge to Social Aims, Pietrasanta, 55045 Lucca, Italy; 3 Department of Psychology, International Telematic University Uninettuno, 00186 Rome, Italy; l.cerniglia@uninettunouniversity.net; 4 Department of Clinical and Dynamic Psychology, Faculty of Medicine and Psychology, Sapienza University of Rome, 00185 Rome, Italy; silvia.cimino@uniroma1.it; 5 School of Psychiatry, University of Pisa, 56100 Pisa, Italy; s.baciard@gmail.com (S.B.); pallucchini.a@gmail.com (A.P.); e.spera@hotmail.it (V.S.); 6 Department of Psychiatry, North-Western Tuscany Region Local Health Unit, Apuan Zone, 54100 Massa, Italy; lucarovai@yahoo.com; 7 Department of Clinical and Experimental Medicine, Section of Psychiatry, University of Pisa, 56100 Pisa, Italy; giulio.perugi@med.unipi.it; 8 G. De Lisio Institute of Behavioral Sciences, 56100 Pisa, Italy; 9 Vincent P. Dole Dual Diagnosis Unit, Department of Specialty Medicine, Santa Chiara University Hospital, University of Pisa, 56100 Pisa, Italy

**Keywords:** psychopathological dimensions, psychopathology of addiction, heroin use disorder, substance use disorder, obesity

## Abstract

*Introduction:* In this study, we used a symptomatology checklist (SCL-90) to substantiate the hypothesis that Substance Use Disorder (SUD) has its own five-dimensional psychopathology. The aim of the present study was to test whether this psychopathology can be differentiated from other psychiatric psychopathological dimensions (such as obesity). *Methods:* The severity and frequency of each of the five dimensions were investigated, at univariate and multivariate levels, by comparing 972 Heroin Use Disorder (HUD) patients (83.5% male, mean age 30.12 ± 6.6, range: 16–59) and 106 obese individuals (50.0% male, mean age 37.59 ± 7.6, range: 24–52). The correlations between the Body Mass Index (BMI) of obese individuals with these psychopathological dimensions were also studied. *Results:* Obese individuals showed higher SCL-90 total scores, global severity index scores, number of items rated positively, and positive symptoms distress index scores than HUD patients. The severity of all psychopathological dimensions was significantly higher in obese individuals. Discriminant analysis showed that Panic-Anxiety and Violence-Suicide severity were more frequent in obese patients, sufficiently so to allow differentiation between HUD (lower severity) and obese individuals (greater severity). At the reclassification level, 70.8% of obese individuals in the sample were reclassified as HUD patients. Psychopathological subtypes characterized by Panic-Anxiety and Violence-Suicide typology were more frequent in obese patients and sufficiently so as to discriminate between groups. Of obese patients, 47.2% were reclassified as HUD patients. The severity of the Worthlessness-Being Trapped dimension was sufficient to predict the BMI of obese individuals. *Conclusions:* Our results suggest that the five-factor psychopathology found in HUD can discriminate between HUD and obese patients, but that there is an area of overlap between the forms of psychopathology found in SUD and those found in obese patients.

## 1. Introduction

A specific psychopathology of Substance Use Disorders (SUD) has recently been proposed. By applying an exploratory principal component factor analysis (PCA) to the Self-Report Symptom Inventory (SCL-90) checklist from a sample of Heroin Use Disorder (HUD) patients completed on entering agonist opioid treatment, it is possible to identify a group of five mutually exclusive psychopathological/psychiatric dimensions: (1) “worthlessness and being trapped”; (2) “somatic symptoms”; (3) “sensitivity-psychoticism”; (4) “panic anxiety”; and (5) “violence-suicide” [1]. The same analyses were performed in parallel to a different sample of HUD patients. The studies led to the identification of the same five-factor solution, which proved not to be substantially influenced by confounding factors such as active heroin use, lifetime psychiatric problems, kind of treatment received or specific drug of abuse (heroin, cocaine or alcohol) [2,3,4,5]. These five psychopathological dimensions appeared to be significantly correlated with the outcome of a variety of agonist opioid treatments [6] and residential treatments [7]. In addition, these five dimensions have demonstrated their capacity to discriminate HUD patients from other psychiatric patients, specifically those affected by major depression [8]. Accordingly, it is possible to speculate that these five dimensions can be used to identify the specific psychopathology of substance use disorder [9].

Obesity is not included in the fifth edition of the Diagnostic and Statistical Manual (DSM-5) as a mental disorder, but there are robust associations between obesity and a number of mental disorders (e.g., binge-eating disorder, depressive and bipolar disorders, schizophrenia) [10]. It has been postulated that obesity is a complex medical condition that may prove to be related, in some way, to addiction [11]. As a result, a “food addiction” model of obesity and overeating has gained increasing popularity in recent years [12,13].

Drug addiction and obesity appear to share several features. Both can be defined as disorders in which the importance attributed to a specific type of reward (food or drug) becomes disproportionate relative to, and at the expense of other types of rewards. Both drugs and food have powerful reinforcing effects, which are partly mediated by abrupt dopamine increases in the brain reward centers. In vulnerable individuals, the sudden dopamine peaks that occur may override the brain’s homeostatic control mechanisms [11,14,15,16]. Some studies have, in fact, confirmed the existence of such alterations during reward processing in obesity, and similarly, in both non-substance and substance addiction [17]. More specifically, neurobiological studies have shown that subjects with either obesity or addictions differed from control subjects in several regions of the brain, including prefrontal areas, subcortical structures and sensory areas. Furthermore, individuals with obesity and SUD exhibited similar types of blood-oxygen-level-dependent fMRI hyperactivity in the amygdala and striatum when processing either generally rewarding stimuli or the more problematic kinds of stimuli (such as food and drug-related stimuli, respectively) [17,18]. While it is evident that the reward circuitry that is impacted by drugs of addiction is prominent in determining food intake, and it is influenced by highly palatable foods and diet-induced obesity, it remains unclear whether these changes can be considered as a type of addiction per se.

The purpose of the present study is to further investigate the validity of the dimensional approach of the SCL-90 in differentiating addiction from other psychopathology by comparing HUD and obese individuals. We expected a differentiation between HUD and obese patients, because even if there are some indications that obesity might be considered a form of addiction [11], it would be incorrect to classify obesity as a type of addiction per se [19].

## 2. Materials and Methods

### 2.1. Design of the Study

Information on patients included in the present study comes from two different datasets. Data regarding HUD patients came from the Pisa Addiction dataset (data collected at treatment entry)—a database including anonymous, individual information originally collected for clinical or other research purposes at the Dual Diagnosis Unit, Santa Chiara University Hospital in Pisa, Italy. Data regarding obese individuals were selected from a five-year dataset of subjects attending Family Caregiving Support Centers working in collaboration with the Department of Clinical and Dynamic Psychology at La Sapienza University in Rome, while also considering their Body Mass Index (BMI). All the subjects recruited for the research had signed an informed consent document; patients evaluated for clinical purposes gave their informed consent for the anonymous use of their clinical data for independent research. Apart from the research protocol, the assessment of these patients was totally non-interventional, so the choice of a subsequent treatment was made in accordance with the conventional criteria that are routinely adopted in clinical practice.

The information obtained was analyzed after implementing a retrospective, naturalistic, cross-sectional comparative design, with a single evaluation of the patients, and with the purpose of estimating the magnitude of differences, in terms of psychopathological symptoms, between patients with HUD and individuals affected by obesity.

### 2.2. Sample

The sample consisted of 1078 patients who fulfilled the diagnosis of obesity, or the DSM-IV, DSM-5 criteria for HUD. None of the patients were undergoing pharmacological, psychiatric or psychological treatment at evaluation time. Of all these patients, 865 (80.2%) were male. The mean age was 30.85 ± 7.0 (range: 16–59).

From the total sample, 972 patients had been diagnosed with HUD and 812 (83.5%) of these were male. The mean age of this group was 30.12 ± 6.6 (range: 16–59).

From the total sample, 106 patients had been diagnosed as obese and 53 (50.0%) of these were male. The mean age of this group was 37.59 ± 7.6 (range: 24–52). Seventy-three (68.9%) patients were classified as Level 1 obese (BMI 30–34); while 33 (31.1%) were Level 2 obese (BMI 35–40). No patients were classified as Level 3 obese (BMI = 40 or more). Obese subjects were not current or past SUD patients. Obese individuals with any other comorbid psychiatric pathologies were also excluded. SCID-I was used to identify and exclude diagnosable DSM-IV disorders from the obesity sample.

### 2.3. Instruments

#### 2.3.1. Self-Report Symptom Inventory (SCL-90)

First developed by Derogatis and colleagues [20], the SCL-90 is made up of 90 items, each rated on a 5-point scale of distress. It is a self-report clinical rating scale oriented toward the symptomatic behavior of psychiatric outpatients. In the case of substance use disorders, the 90 SCL items reflect five primary symptom dimensions believed to underlie the large majority of symptom behaviors that are observed in this class of patients. The primary symptom dimensions are: Worthlessness-Being Trapped, Somatic Symptoms, Sensitivity-Psychoticism, Panic Anxiety, Violence-Suicide [1].

These five dimensions have been empirically established and primarily validated on a sample involving over 2500 substance use disorder patients [4,5,21]. On the basis of the highest z scores obtained on the five SCL-90 dimensions, subjects can be assigned to one of five mutually exclusive groups.

A brief description of the symptom constructs defined by these dimensions follows below.

**Worthlessness-Being Trapped**—reflects a broad range of the concomitants of the clinical depressive syndrome. This dimension mirrors feelings of worthlessness and of being trapped or caught. Patients feel lonely, blue, hopeless about the future, with no interest in things; they feel lonely even when with other people, inferior to others, blocked in getting things done, tense or keyed up, nervous when left alone. They are thinking everything is an effort, and they feel guilty. They easily feel hurt. They never feel close to another person. They find it difficult to concentrate, and have the idea that something is wrong with their mind. They are worried about sloppiness or carelessness, worried too much about things; they show loss of sexual interest or pleasure, think their mind is going blank, blaming themselves for things. They report unwanted thoughts, words, or ideas that will not leave their mind. This dimension does not include being concerned about thoughts of death or suicidal ideation.

**Somatic Symptoms**—reflects distress arising from perceptions of bodily dysfunction. Patients are characterized by muscle soreness, feelings of heaviness in their arms or legs, having hot and cold spells, nausea or upset stomach, trouble falling asleep, feeling weak in parts of their body, with pain in the lower back, feeling low in energy or slowed down. Their sleep is restless or disturbed, and they wake up early in the morning. They report numbness or tingling in parts of their body, a lump in their throat, trembling, their heart pounding or racing, trouble in getting their breath, poor appetite, pains in heart or chest, while easily feeling annoyed and irritated.

**Sensitivity-Psychoticism**—focuses on feelings of a full continuum of psychotic behaviors. Patients report feeling that they are watched or talked about by others, that other people are aware of their private thoughts, that people are unfriendly or dislike them, that others do not understand them or are unsympathetic, that someone else can control their thoughts, that they have thoughts that are not their own. They feel uneasy when people are watching or talking about them, and very self-conscious in the company of others. They must check and double check what they are doing or have to do things very slowly to ensure correctness. They feel that people will take advantage of them if they let them, that others are not giving them proper credit for their achievement. They feel uneasy in crowds, for example when they are out shopping or at a movie, having ideas or beliefs that others do not share. They feel uncomfortable about eating or drinking in public, and feel pushed to get things done; they think that familial matters are strange or unreal.

**Panic-Anxiety**—subsumes a set of symptoms and experiences usually associated clinically with a high level of manifest anxiety. Patients report feelings of being afraid in an open space or on the streets; afraid to go out of their house alone, afraid to travel on buses, subways, or on trains; afraid they will faint in public. They experience spells of terror or panic, faintness or dizziness, feelings of fear. They suddenly feel scared for no reason and have to avoid certain things, places, or activities because they feel frightened by them.

**Violence-Suicide**—this dimension is organized around three categories of hostile behavior: thoughts, feelings, and actions; it also comprises thoughts of death and suicidal ideation. Patients report the urge to shout or to throw things, to break or smash things up, to beat, injure or harm someone. They have often had outbursts of bad temper that they could not control, they have been impelled to get into frequent arguments, feeling so restless they could not sit still, with nervousness or shakiness inside. At the same time, they have had thoughts of ending their life, of death or dying.

The three global scores usually calculated from the SCL-90 items are: Total SCL-90 score (sum of all items); the number of items rated positively (PST); and the positive symptom distress index (PSDI), which is calculated by dividing the sum of all items by the score for PST.

#### 2.3.2. Psychiatric Diagnostic Evaluation. Structured Clinical Interview for DSM-IV Axis I Disorders (SCID-I), Clinician Version

This instrument [22] helps clinicians to make standardized, reliable, and accurate diagnoses and avoid the common problem of “premature closure” (a premature focus on one diagnostic possibility). Specifically adapted from the research standard for Axis I structured clinical interviewing for use in clinical settings, the SCID-I covers the DSM-IV diagnoses most commonly seen by clinicians and includes the diagnostic criteria for these disorders with corresponding interview questions. The SCID-I is divided into six self-contained modules that can be administered in sequence: mood episodes; psychotic symptoms; psychotic disorders; mood disorders; substance use disorders; and anxiety, adjustment, and other disorders.

### 2.4. Data Analysis

In this study, HUD and obese individuals were compared for demographic and psychopathological dimensions by means of the chi-squared test, with Bonferroni’s correction, for categorical variables, and Student’s *t*-test for continuous variables.

Differences in severity of psychopathological dimensions were analyzed, at multivariate level, by means of discriminant analysis. To select the prominent characteristics of HUD patients and in order to take into account possible confounding factors, a logistic forward stepwise regression analysis was used, considering the presence of a HUD diagnosis as criterion, and belonging to one of the five psychopathological groups, along with age, gender and severity of symptomatology as predictors. Statistical routines of SPSS (v20.0, IBM Corp, New York City, NY, USA) were used.

## 3. Results

HUD patients were younger (30.12 ± 6.6 years) than the obese subjects (37.59 ± 7.6 years). This difference was statistically significant (*t* = −10.82; *p* < 0.001). Males were more frequent among HUD patients (*N* = 812; 83.5%) than among obese individuals (*N* = 53; 50.0%) (chi-squared = 67.81; *p* < 0.001).

Table 1 reports psychopathological severity differences at univariate and multivariate level. Obese individuals showed higher total SCL-90 scores, global severity index scores, number of items rated positively and positive symptoms distress index scores than HUD patients. The severity of all psychopathological dimensions was significantly higher in obese individuals. Discriminant analysis showed that Panic-Anxiety and Violence-Suicide severity was sufficient to differentiate between HUD (lower severity) and obese individuals (greater severity), whereas Worthlessness-Being Trapped, Somatic Symptoms and Sensitivity-Psychoticism did not differentiate. At the reclassification level, 95.7% of HUD patients were confirmed, while 70.8% of obese individuals were reclassified as HUD patients. In summary, severity of psychopathology as a leading parameter allows the differentiation of HUD patients from obese ones.

Psychopathological subtypes characterized by Worthlessness-Being Trapped’, Somatic Symptoms and Sensitivity-Psychoticism symptomatology were more frequent among HUD patients, whereas Panic-Anxiety and Violence-Suicide symptomatology were more frequent among obese individuals (Table 2). At multivariate level, after checking the analysis for age, gender, and severity of symptomatology, the predominant characteristic of obese individuals was the Panic-Anxiety (OR = 2.98) or the “Violence-Suicide” (OR = 34.09) psychopathological subtype (Table 3). At the reclassification level, after correction for age and gender, 47.2% of obese individuals were reclassified as HUD patients. Typology of psychopathology can also differentiate HUD patients from obese ones.

The Body Mass Index of obese individuals was positively correlated with the severity of Worthlessness-Being Trapped (r = 0.37; *p* < 0.001), Panic-Anxiety (r = 0.34; *p* < 0.001), Sensitivity-Psychoticism (r = 0.28; *p* < 0.05), Somatic Symptoms (r = 0.21; *p* < 0.05), and Violence-Suicide (r = 0.21; *p* < 0.05) dimensions. At multivariate level, the severity of the Worthlessness-Being Trapped dimension (β = 0.37; *t* = 4.12; *p* < 0.001) was sufficient to predict the BMI of obese individuals (F = 17.02; *p* < 0.001).

## 4. Discussion

In this study, the severity and typology of psychopathological symptoms were enough to permit differentiation between HUD and obese individuals. A considerable percentage of obese subjects show similarities with HUD patients. BMI was positively correlated with the five psychopathological dimensions, and also with the severity of the Worthlessness-Being Trapped dimension—this is the most important dimension found in SUD patients [1], and was sufficient to predict the BMI of obese individuals. In summary, as expected, the severity and typology of the psychopathology found in HUD patients, is what allows discrimination between HUD and obese patients. Nevertheless, significant numbers of obese individuals show the same psychopathology as HUD patients. This percentage is higher (70.8%) if we consider only psychopathological symptoms or lower (47.2%) if we consider other confounding variables such as age, gender and severity of psychopathology. BMI was shown to be linked with a specific psychopathology of SUD.

The relationship between addiction, overeating (either chronically or in binge episodes), and obesity has been approached from different perspectives. The conceptualization of obesity as a form of addiction is a major issue which has led to the birth of a new term to define this behavior: Food Addiction (FA), describing a behavioral phenotype of food consumption mirroring the clinical criteria for substance dependence [23]. Many authors have, however, questioned the continued use of the term Food Addiction as controversial and view it as a potentially flawed way of describing the behavior of overeating [17,24]. In fact, a growing number of studies suggest that obesity and addiction may have some parallels with respect to the neural response to rewarding stimuli [25]. The “food addiction” hypothesis postulates that exposure to highly palatable foods alters the brain’s reward circuitry, driving an addiction-like behavioral phenotype of compulsive overeating [26,27,28,29]. Changes in dopamine and opioid receptor binding, enkephalin mRNA expression and dopamine and acetylcholine release in the nucleus accumbens would explain the behavioral features that are shared by addiction (to food high in sugar calories) and obesity, as happens with bingeing, withdrawal, craving, and sensitization [30,31,32,33,34]. However, a low prevalence of FA in obese subjects has been found in one study, suggesting that eating of an addictive type is unlikely to be a causal mechanism for most people with obesity [35].

Clearly, binge-eating disorder (BED) is associated with being overweight and obesity [36,37] and obesity is associated with BED [38]. Unlike obesity, however, BED is currently categorized formally as a mental illness [10]. Recurrent binge-eating episodes can, in fact, be considered as toxicomanic behaviors pointing to a “food addiction” [39]. Previous investigations have found that only around 50% of obese individuals diagnosed with BED reached the “food addiction” threshold when specific questionnaires were used, thus indicating that there is no specific overlap between these clinical conditions [40]. The borders separating BED, food addiction and obesity are hazy. While binge eating has been conceptualized as a form of addictive behavior, it is not a major cause of excessive eating because binge eating has a far lower prevalence than obesity [19]. Moreover, FA does not appear to be a major cause of the excessive eating that is responsible for obesity [19]. The latest studies consider BED and food addiction as occurring with a relatively low probability in the general population, whereas about a third of the adult population in many Western countries is obese. While it has been argued that it is actually excessive to model obesity as an addiction disorder; this argument can be countered by the fact that, even if many people are obese, few have a legitimate psychobiological pathology [41], and only a subset of obese people display any of the behavioral characteristics that are associated with addiction. Earlier studies pointed out that only about 10–25% of overweight and obese individuals with addiction-like behavior meet the Yale Food Addiction Score criteria [27]. One of the strengths of the present study is its selection of obese individuals without any other mental illness, thus ensuring that the presence of psychopathology related to SUD is correlated with obesity per se, not with the presence or absence of BED. As Gearhardt et al. suggest, the concept of food addiction appears to capture clinically relevant information in participants who do not meet the criteria for BED [42]. If obesity could be related in any way to addiction, the five-factor psychopathological structure found in HUD patients would not be able to definitively differentiate HUD patients from obese ones. This would be particularly true (and it is probably what happened in the present study) in those with food addiction that showed the same psychopathological dimensions of HUD patients.

In line with our findings, there is evidence on psychopathological grounds showing a higher ratio of various psychopathology (depression, behavioral problems, low esteem) among clinically obese adolescents than among non-clinically obese adolescents [43]. To date, few findings are available on food addiction symptomatology and obesity, especially if we look at the role of BMI. At a psychopathological level, we found a positive correlation between BMI and the severity of the five psychopathological dimensions. Interestingly, researchers usually assume linear relationships between variables and only a few studies have examined non-linear relationships between BMI and eating-related constructs (e.g., reward sensitivity) [44,45]. Studies that calculated correlations between BMI and food addiction symptoms or compared groups of food-addicted vs. non-addicted individuals for BMI did not yield evidence of a clear positive relationship between BMI and food addiction [45]. There seems to be only a marginally positive relationship between binge eating severity and BMI in samples with a wide body mass range, and there is no such relationship within samples of obese individuals [45]. Other studies comparing groups with and without food addiction or BED usually fail to find any differences in BMI, even when considering variables such as age, race, or gender between obese participants with and without FA [46].

The present study has several limitations: (i) the retrospective methodology; (ii) no rating scales designed to inquire into the category of “food addiction” (e.g., Yale Food Addiction) were administered to obese individuals; (iii) no statement can be made about the prevalence of possible Axis-II disorders in this sample; (iv) no diagnoses of eating disorders were investigated in the HUD sample; and (v) the considerable differences in sample size and composition may have had a negative impact on the power of the applied statistical tests.

## 5. Conclusions

The specific five-factor psychopathology of HUD is able to discriminate between HUD patients and obese patients, but a significant percentage of obese individuals show the same set of psychopathological dimensions as HUD individuals, suggesting that there may be some overlap between psychopathology in cases of SUD and obesity. Further studies are now needed to determine if the high level of obese patients reclassified in our study as HUD patients, is due to the presence in our sample of patients with food addiction.

## Figures and Tables

**Table 1:** Psychopathological severity in Heroin Use Disorder (HUD) patients and obese individuals.

Variable	HUD Patients *N* = 972	Obese Individuals *N* = 106			
	M ± sd	M ± sd	*t*	*p*	
Total SCL-90 severity	89.85 ± 53.7	158.19 ± 43.5	−12.65	<0.001	
Global Severity Index	1.00 ± 0.6	1.76 ± 0.5	−12.56	<0.001	
PST, number of	48.79 ± 18.5	66.18 ± 15.2	−9.31	<0.001	
PSDI severity	1.73 ± 0.5	2.39 ± 0.3	−12.89	<0.001	
Psychopathological dimensions					DF
1-Worthlessness-Being Trapped	1.22 ± 0.8	2.00 ± 0.5	−10.42	<0.001	
2-Somatic Symptoms	1.29 ± 0.8	1.87 ± 0.7	−7.93	<0.001	
3-Sensitivity-Psychoticism	0.83 ± 0.6	1.46 ± 0.5	−9.62	<0.001	
4-Panic Anxiety	0.46 ± 0.6	1.25 ± 0.8	−12.55	<0.001	0.63
5-Violence-Suicide	0.98 ± 0.7	1.93 ± 0.7	−12.51	<0.001	0.62
*Centroids*	−0.36	0.87			

HUD = Heroin Use Disorder; PST = positively rated items (number of); PSDI = positive symptom distress index; DF = Discriminant Function; Statistics: Lambda = 0.84; F = 67.95: *p* = <0.001; 95.7% of HUD patients were confirmed; 70.8% of obese individuals were re-classified as HUD patients.

**Table 2:** Psychopathological typology in Heroin Use Disorder (HUD) patients and obese patients.

Variable	HUD Patients *N* = 972	Obese Individuals *N* = 106		
	*N* (%)	*N* (%)	Chi	*p*
Psychopathological Subtypes				
1-Worthlessness-Being Trapped	142 (14.6)a	07 (06.6)b		
2-Somatic Symptoms	329 (33.8)a	20 (18.9)b		
3-Sensitivity-Psychoticism	215 (22.1)a	06 (05.7)b		
4-Panic Anxiety	170 (17.5)a	34 (32.1)b		
5-Violence-Suicide	116 (11.9)a	39 (36.8)b	75.51	<0.001

**Table 3:** Logistic regression analysis. Criterion: being in the obese group. Predictors: Psychopathological typology corrected for age, gender, and severity of symptomatology (only significant results are reported in the table).

STEP	Variable	Odds Ratio	95% CI	*p*
1	Total SCL-90	1.02	1.02–1.03	<0.001
2	Age	1.18	1.13–1.23	<0.001
3	Psychopathological subtype			
	1-Worthlessness-Being Trapped	1.00		
	4-Panic Anxiety	2.98	1.02–8.72	0.046
	5-Violence-suicide	34.09	11.15–104.18	<0.001
4	Female gender	11.21	5.92–21.22	<0.001

Statistics: chi squared 346.07, df 7, *p* < 0.001; 47.2% of obese individuals were re-classified as HUD patients.
